# Estimation of Total Body Skeletal Muscle Mass in Chinese Adults: Prediction Model by Dual-Energy X-Ray Absorptiometry

**DOI:** 10.1371/journal.pone.0053561

**Published:** 2013-01-07

**Authors:** Xinyu Zhao, ZiMian Wang, Junyi Zhang, Jianming Hua, Wei He, Shankuan Zhu

**Affiliations:** 1 Obesity and Body Composition Research Center, Chronic Disease Research Institute, School of Public Health, Zhejiang University, Hangzhou, China; 2 Obesity Research Center, St. Luke’s–Roosevelt Hospital Center, Columbia University College of Physicians and Surgeons, New York, New York, United States of America; 3 Department of Statistics, Columbia University, New York, New York, United States of America; 4 Department of Radiology, Second Affiliated Hospital of Zhejiang University, Hangzhou, China; 5 Department of Nutrition and Food Hygiene, School of Public Health, Zhejiang University, Hangzhou, China; The University of Tennessee Health Science Center, United States of America

## Abstract

**Background:**

There are few reports on total body skeletal muscle mass (SM) in Chinese. The objective of this study is to establish a prediction model of SM for Chinese adults.

**Methodology:**

Appendicular lean soft tissue (ALST) was measured by dual energy X-ray absorptiometry (DXA) and SM by magnetic resonance image (MRI) in 66 Chinese adults (52 men and 14 women). Images of MRI were segmented into compartments including intermuscular adipose tissue (IMAT) and IMAT-free SM. Regression was used to fit the prediction model 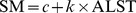. Age and gender were adjusted in the fitted model. The piece-wise linear function was performed to further explore the effect of age on SM. ‘Leave-One-Out Cross Validation’ was utilized to evaluate the prediction performance. The significance of observed differences between predicted and actual SM was tested by *t* test and the level of agreement was assessed by the method of Bland and Altman.

**Results:**

Men had greater ALST and IMAT-free SM than women. ALST was the primary predictor and highly correlated with IMAT-free SM (R^2^ = 0.94, SEE = 1.11 kg, P<0.001). Age was an additional predictor (SM prediction model with age adjusted *R* ^2^ = 0.95, SEE = 1.05 kg, *P*<0.001). There was a piece-wise linear relationship between age and IMAT-free SM: IMAT-free SM = 1.21×ALST−0.98, (Age <45 years) and IMAT-free SM = 1.21×ALST−0.98−0.04× (Age−45), (Age ≥45years). The prediction performance of this age-adjusted model was good due to ‘Leave-One-Out Cross Validation’. No significant difference between measured and predicted IMAT-free SM was detected.

**Conclusion:**

Previous SM prediction model developed in multi-ethnic groups underestimated SM by 2.3% and 3.4% for Chinese men and women. A new prediction model by DXA has been established to predict SM in Chinese adults.

## Introduction

Skeletal muscle is the largest non-adipose tissue component of the tissue-system level of human body composition [1] and is central to the study of nutritional, physiologic and metabolic processes [2], [3], [4]. Magnetic resonance imaging (MRI) is currently applied as the golden standard for evaluating skeletal muscle due to its high accuracy and lack of radiation to the subjects [4], [5]. However, the high cost of MRI precludes its routine use in research and clinical practice. Presently, dual energy X-ray absorptiometry (DXA) is considered an alternative approach to estimate skeletal muscle *in vivo,* with substantially lower costs and less radiation exposure [4], [6]. So far, several studies have used DXA to predict skeletal muscle mass [6], [7], [8], [9], [10], [11].

Quantification of skeletal muscle mass significantly contributes to further understanding the characteristics of skeletal muscle, instructing the measurement and evaluation of physique and improving national physical fitness. China is the most populous country in the world, accounting for nearly 20% of the world’s population. However, there is no reported DXA-based skeletal muscle prediction model applicable to Chinese adults.

The prediction model from Kim *et al.* by DXA was developed and validated through subjects of multiple ethnicities, including Caucasians, African-Americans, Hispanics and Asians [10]. However, the interpretation and application of this prediction model in Chinese adults are questionable. First, the Asian subjects in Kim’s study were a mixture of multiple Asian populations. In addition to China, subjects were also recruited from India, Korea and Japan and other Asian countries. Previous studies have revealed that there were differences in body composition among people from different Asian countries [12]. Second, there may be differences in body composition between the Asian population living in the U.S. and those in their originating countries [13], [14]. Third, the Asian subjects in Kim’s study only accounted for 12.8% and 7.5% of the development and validation models, respectively. The established model was validated only in 7 Asians, and the quantitative difference between the proportions of Asian subjects in the development and validation groups are too great to conclude that ethnicity will not affect the amount of total body skeletal muscle predicted by DXA.

The objective of the present study is to establish a DXA-based skeletal muscle prediction model in Chinese adults by using MRI as the reference standard.

## Methods

### Protocol

From the perspective of body composition, appendicular tissues primarily consist of skeletal muscle, bone, fat, skin and connective tissue [6]. Appendicular lean soft tissue (ALST), measured by DXA, is a fat- and bone mineral-free tissue which consists of skeletal muscle, skin, connective tissue, and tendons [8], [15], [16], [17]. Because a large proportion (∼75%) of total body skeletal muscle exists in appendicular tissues [18] and a large proportion of ALST is SM, ALST is considered as a measure of appendicular SM (ASM) [7].

**Figure 1:**
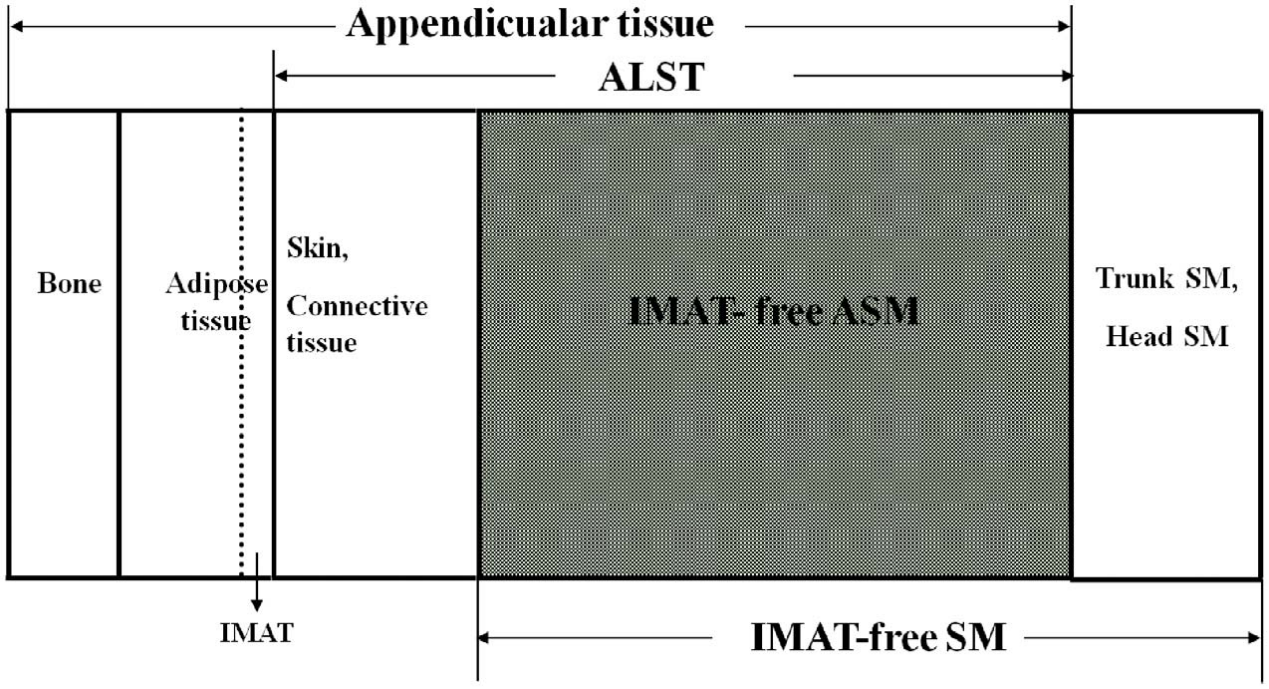
Relationship between appendicular tissue, appendicular lean soft tissue (ALST), adipose tissue, bone, intermuscular adipose tissue (IMAT), IMAT-free appendicular skeletal muscle (IMAT-free ASM) and IMAT-free skeletal muscle (IMAT-free SM).

A small amount of adipose tissue that locates between muscle groups and beneath the muscle fascia is defined as intermuscular adipose tissue (IMAT) [19]. The association among appendicular tissue, ALST, ASM, SM and IMAT is illustrated in **Figure 1**. In the current study, total body skeletal muscle mass (SM) is defined as the skeletal muscle with IMAT. The association among IMAT, IMAT-free SM and SM is represented as:
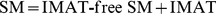
(1)


### Subjects

A total of 68 subjects aged from 20 to 80 years were voluntarily recruited through leaflets and posters provided by the Obesity and Body Composition Research Center (OBCRC), Zhejiang University School of Public Health between December 2009 and July 2010. The study was approved by the Ethics Committee of the Second Affiliated Hospital, Zhejiang University. Written consent was obtained from all participating subjects prior to testing. None of the individuals were on medication or participated in vigorous physical activity. One subject was excluded because of BMI <18.5 kg/m^2^. Another subject was excluded because one highly influential sample with cooks distance was close to 1.0 and absolute value of DFFITS was close to 2 [20], [21]. Therefore, 66 subjects (52 men and 14 women) were finally included in the analysis.

### Body Composition Analysis

Subjects underwent three evaluations within one day: anthropometry and DXA were performed at OBCRC, and MRI was performed at the Second Affiliated Hospital of Zhejiang University, China.

#### Anthropometry

Weight and height were measured while barefoot and wearing light clothing. Body weight was measured to the nearest 0.1 kg (Detecto, USA). Height was measured with a hypsometer to the nearest 0.1 cm. Body mass index (BMI) was calculated as weight in kilograms divided by height in meters squared.

#### Dual-energy X-ray absorptiometry

DXA (GE Lunar Prodigy, WI, USA with software version 11.40.004) was implemented to measure lean soft tissue, fat and bone mineral in both whole body and specific regions of interest. By using specific anatomic landmarks, legs, arms and trunk were isolated on the anterior view planogram using the DXA system’s automated software. The DXA software then provided compositional estimates of the legs, arms, trunk, head and whole-body. ALST was calculated as the sum of lean soft tissue in the right and left legs and arms. DXA was calibrated daily against a phantom with the manufacturer’s precision standards of ≤0.8%.

#### Magnetic resonance imaging

Whole-body MRI imaging was performed by a 3.0 Tesla MRI scanner (Signa, GE Healthcare, USA). Subjects were required to lie in the magnet bore in a supine position with arms extended overhead. A T1 weighted, spin-echo, axial plane sequence was performed with a 1500 millisecond repetition time and a 17 millisecond echo time. Transverse images (10 mm image thickness) were obtained every 50 mm from hand to foot [22]. The intervertebral space between the fourth and fifth lumbar vertebrae (L4–L5) was used as the point of origin. Three series of seven images were obtained for the lower body and three series of seven images were obtained for the upper body. The protocol involved the acquisition of 36–45 images, depending on the height of the subject.

**Figure 2:**
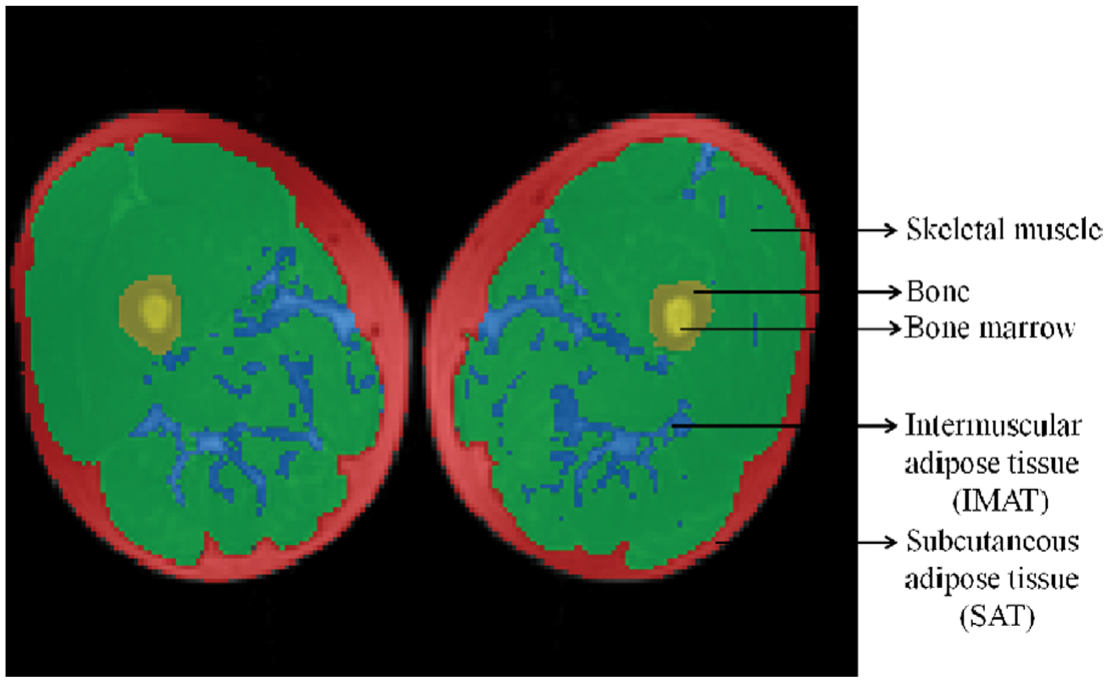
Segmentation of the original T1- weighted magnetic resonance image of legs. Subcutaneous adipose tissue (SAT), intermuscular adipose tissue (IMAT), skeletal muscle, bone and bone with marrow are shown.

The SliceOmatic 4.3 software (TomoVision Inc, Montreal, Canada) was applied to segment tissue compartments. IMAT-free SM and IMAT were segmented automatically first by the combination of functions offered by the software including threshold, region growing, mathematic morphology and snakes. We then adjusted manually and calculated their cross-section areas. Segmentation of IMAT and skeletal muscle on MRI image is indicated in **Figure 2** **.** Volumes of IMAT-free SM and IMAT by MRI were calculated as 
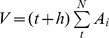
, where 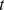 is the thickness (10 mm) of each image, 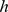 (40 mm) is the distance between consecutive images and 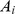 is each image’s cross sectional area [23]. Volumes were converted to mass by multiplying the density of 1.04 kg/l for IMAT-free SM and 0.92 kg/l for IMAT [18]. Our study shows that the technical error for repeated measurement of the same scan by the same observer of IMAT-free SM and IMAT volumes are 1.0% and 0.9%, respectively.

### Statistical Analysis

Data was grouped by gender and summarized in terms of mean ± standard deviations (SD). The differences in the attributes of men and women were tested by Student’s *t* test. Pearson’s correlation was performed to explore the associations between DXA-measured ALST and MRI-measured SM. A simple linear regression model was first applied to the data as: 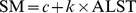, where 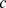 is the intercept and 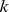 is the slope of the equation. Then other independent variables such as age and gender were considered as the adjustment to the simple linear regression model. The adjusted *R^2^* and standard error of estimate (SEE) were used to evaluate model-fitting performance.

To evaluate the models’ performance via out of sample prediction, we applied the method of ‘Leave One Out Cross Validation’ [24] for regression models, which is a rotation analysis of prediction performance based on N–1 training samples and 1 validation sample where N is the total sample size. Specifically, the original data set was partitioned into a training set of N–1 samples and a validation set of the remaining one. A regression model was fitted to the training set. Based on the fitted model, a prediction was made for the validation sample and the predicted residual was calculated. This procedure was replicated N times for all possible partitions and then the prediction performance was summarized as Coefficients of Variation (CV) statistics. In the present study, the root mean square error of the predicted residuals was used to compare the out of sample prediction performance for potential models. In addition, final model was also checked by both F-test and permutation test for analysis of variance (ANOVA).

Pearson correlation was also explored between predicted SM values and the corresponding actual values measured by MRI. The difference and the agreement between total body SM measured by MRI and predicted from the equation were tested by Student’s *t* test and the method of Bland-Altman plot [25].

All statistical analyses were performed by Stata software (version 11.0 for Windows; Stata Corporation, College Station, TX) and statistical significance level was set at *P*<0.05 (two-tailed).

## Results

### Characteristics and Body Composition

The characteristics and body composition of 66 subjects (52 men and 14 women) are presented in **Table 1**. There were no significant differences in age and BMI between men and women. Men tended to have a greater body mass, height, ALST, IMAT, IMAT-free SM and SM than women. In contrast, women had a greater percentage of body fat than men.

**Table 1:** Subject characteristics and body composition.

	Men	Women	*P*
**n**	52	14	––
**Age** (year)	52.0±13.3 (28–79)	46.6±11.8 (30–72)	0.17
**Body mass** (kg)	67.6±10.2 (52.4–90.6)	56.3±9.3 (43.9–75.5)	<0.001
**Height** (cm)	167.0±6.7 (150.4–181.6)	157.1±4.9 (148.2–165.7)	<0.001
**BMI** (kg/m^2^)	24.2±3.1 (18.5–32.2)	22.7±2.7 (18.4–27.5)	0.11
**%Fat**	22.2±5.7 (7.3–36.0)	30.4±3.6 (21.6–35.8)	<0.001
**ALST** (kg)	21.7±2.8 (16.4–28.7)	15.3±2.5 (12.1–19.9)	<0.001
**IMAT** (kg)	0.6±0.2 (0.2–1.3)	0.5±0.1 (0.3–0.7)	0.04
**IMAT-free SM** (kg)	25.0±3.6 (18.4–34.5)	17.4±3.4 (13.2–23.5)	<0.001
**SM** (kg)	25.7±3.7 (19.0–35.9)	18.0±3.4 (13.6–24.1)	<0.001

Values are means ± SD, with range in parentheses.

**Abbreviations**: BMI, body mass index; %Fat, percentage of body mass as fat; ALST, appendicular lean soft tissue;

IMAT, intermuscular adipose tissue; SM, total body skeletal muscle.

### Evaluation of the SM Prediction Model by Kim et al. in Chinese Adults

**Figure 3:**
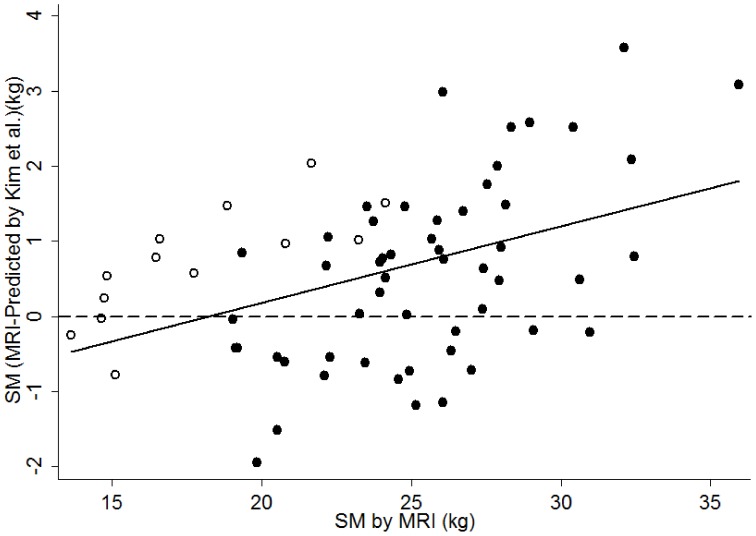
Difference of total body skeletal muscle (SM) by MRI and by Kim *et al*. on the ordinate versus SM by MRI on the abscissa in men (•, n = 52) and women (○, n = 14). The correlation between the difference of SM by MRI and by Kim *et al.* and SM by MRI was 0.57 (SEE = 1.03 kg, *P*<0.001) in men and 0.67 (SEE = 0.62 kg, *P*<0.001) in women. The reference line (y = 0) is shown.

By substituting the values of ALST, age and gender into the SM prediction model by Kim *et al.* [10], SM predicted according to Kim’s equation was significantly less than that measured by MRI which was underestimated by 2.3% and 3.4% in men and women, respectively (**Table 2**). There was a significant bias between the difference of SM measured by MRI and calculated by the prediction model of Kim *et al.* (*r* = 0.43, *P*<0.001) and two thirds (44/66, 67%) of the subjects were underestimated from Kim’s prediction equation (**Figure 3**)**.**

**Table 2:** Comparison of SM measured by MRI and predicted by Kim’s equation [10].

	SM (kg) by MRI	SM (kg) by Kim *et al.*	Difference	*P*
**All men** (n = 52)	25.7±3.7	25.1±3.2	0.59±1.24	0.0013
**All women** (n = 14)	18.0±3.4	17.4±2.9	0.62±0.80	0.013
**Men+Women**				
**<45 years** (n = 23)	24.4±4.7	23.8±4.4	0.60±1.13	0.019
**≥45 years** (n = 43)	23.8±4.9	23.3±3.5	0.59±1.18	0.002
**Total** (n = 66)	24.0±4.8	23.4±4.4	0.59±1.15	<0.001

Values are means ± SD.

**Abbreviation:** SM, total body skeletal muscle.

### Prediction Model of IMAT-free SM

#### Model development

**Figure 4:**
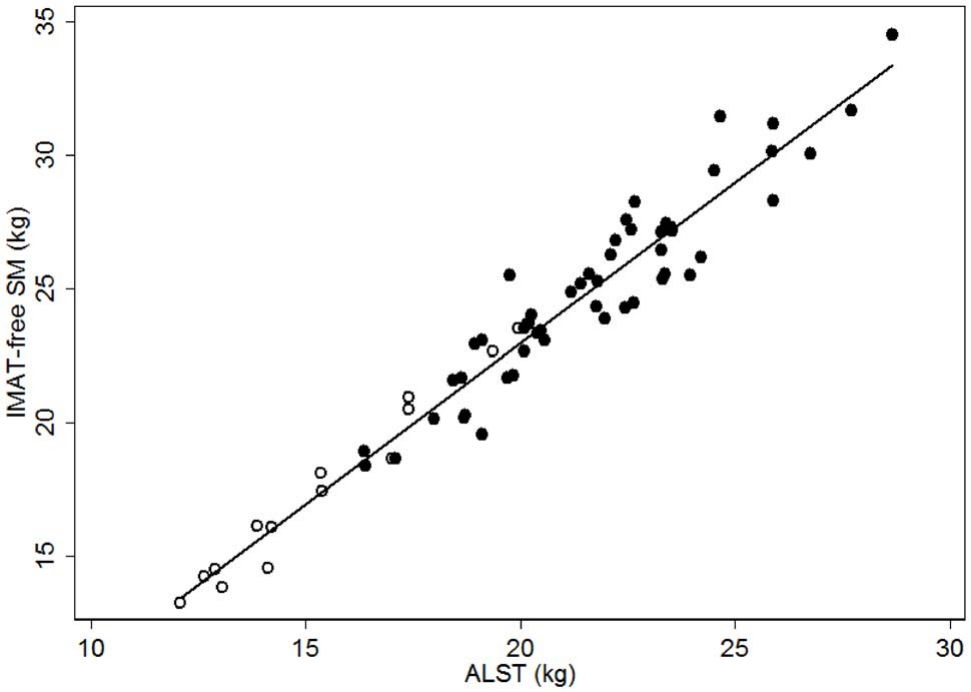
Intermuscular adipose tissue free total body skeletal muscle (IMAT-free SM) measured by MRI on the ordinate versus appendicular lean soft tissue (ALST) measured by DXA on the abscissa. The correlation between IMAT-free SM and ALST in men (•, n = 52) and women (○, n = 14) are *r* = 0.94 and *r* = 0.98, respectively. IMAT-free SM = 1.20×ALST−1.13; *R^2^* = 0.94, SEE* = *1.11 *kg,* n = 66.

The ratio of IMAT-free SM to ALST was 1.15±0.06 in men and 1.13±0.05 in women with the corresponding CVs of 5.2% and 4.4%, respectively. There was a significant correlation between IMAT-free SM and ALST (*r = *0.97, *P*<0.001) (**Figure 4**).

Developed models for predicting IMAT-free SM from multiple linear regression analyses are presented in **Table 3** **.** ALST (in kg) was the strongest predictor of IMAT-free SM (in kg), explaining 94.3% of the between-subject variance with a SEE of 1.11 kg.
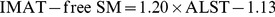
(2)


**Table 3:** Developed equations for predicting total body skeletal muscle mass.

Dependent variable		Independent variables				
	Model	ALST	Age (≥45 years)	Intercept	Adjusted *R* ^2^	SEE (*kg*)
	1	1.20±0.04		−1.13±0.76	0.94	1.11
**IMAT-free SM**		(*P*<0.001)				
	2	1.21±0.03	−0.04±0.01	−0.98±0.72	0.95	1.05
		(*P*<0.001)	(*P = *0.0053)			
	1	1.24±0.04		−1.14±0.78	0.94	1.15
**SM**		(*P*<0.001)				
	2	1.25±0.04	−0.04±0.01	−1.00±0.75	0.95	1.09
		(*P*<0.001)	(*P = *0.0074)			

Values are means ± SD.

**Abbreviations**: ALST, appendicular lean soft tissue; IMAT, intermuscular adipose tissue; SM, total body skeletal muscle.

No significant interaction effect was observed between gender and ALST (F = −0.84, *P* = 0.41). The adjustment of gender failed to contribute to the developed model (*P* = 0.57). The inclusion of age together with ALST in the multiple linear regression model contributed 0.3% to the variance of IMAT-free SM. To further examine the effect of age on IMAT-free SM, we modeled the age effect (adjustment) as a piece-wise linear function which was remaining constant for the middle-aged and decreasing for the elderly people. The cutoff point of the piece-wise linear effect of age was estimated by minimizing the SEE of the following model. The cut-off point turned out to be 45 years old for the database.
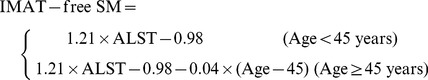
(3)


The inclusion of the age effect together with ALST in the multiple regression models captured an additional 0.6% of the variance in measured IMAT-free SM mass (**Table 3**). With the age increase, subjects older than 45 years contributed a negative effect in IMAT-free SM (Coefficient = −0.04, *P* = 0.0053).

**Figure 5:**
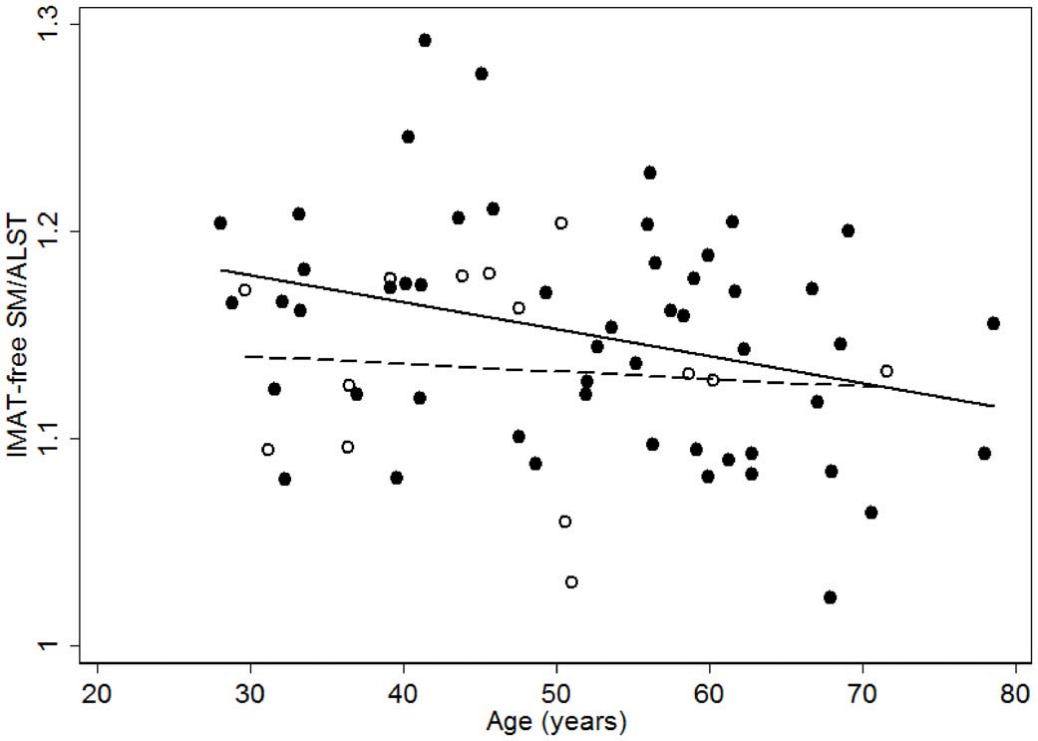
Ratio of IMAT-free SM to ALST (IMAT-free SM/ALST) on the ordinate versus age on the abscissa in men (•) and women (○). Men : IMAT-free SM = −.0.001× age +1.22 *r* = −0.31, *P = *0.02; n = 52. Women : IMAT-free SM = −.0.004×age +1.14 *r* = −0.09, *P = *0.77; n = 14.

In addition, the ratio of IMAT-free SM to ALST was 1.18±0.06 in men and 1.17±0.05 in women with the corresponding CVs of 5.1% and 4.3%, respectively. IMAT-free SM/ALST significantly decreased with age in men (IMAT–free SM = -0.001×age +1.22, *P* = 0.02 ) but not in women (IMAT–free SM = −.0.001×age +1.22, *P = *0.77 ) as indicated in **Figure 5**.

#### Model-validation

Prediction performance was compared the default model 1 (equation 2) and age-adjusted model 2 (equation 3) via ‘delete-one (leave one out) cross validation’ on the root mean squared error of predicted residuals. The root mean squared errors for these two models based on the whole sample are 1.10 and 1.03 kg respectively. The root mean squared errors for the “leave one out cross validation” are 1.13 kg for the default model and 1.08 kg for the age-adjusted model. The age-adjusted model is also favorable due to out of sample prediction performance. The cut-off point of the piece-wise linear age effect was also verified by delete-one cross validation. Apart from the “leave-one-out” validation technique, the final prediction model with age and ALST adjusted, was also checked by both F-test and permutation test for analysis of variance (ANOVA). The testing results showed consistent evidence that both ALST and age effect are significant.

**Figure 6:**
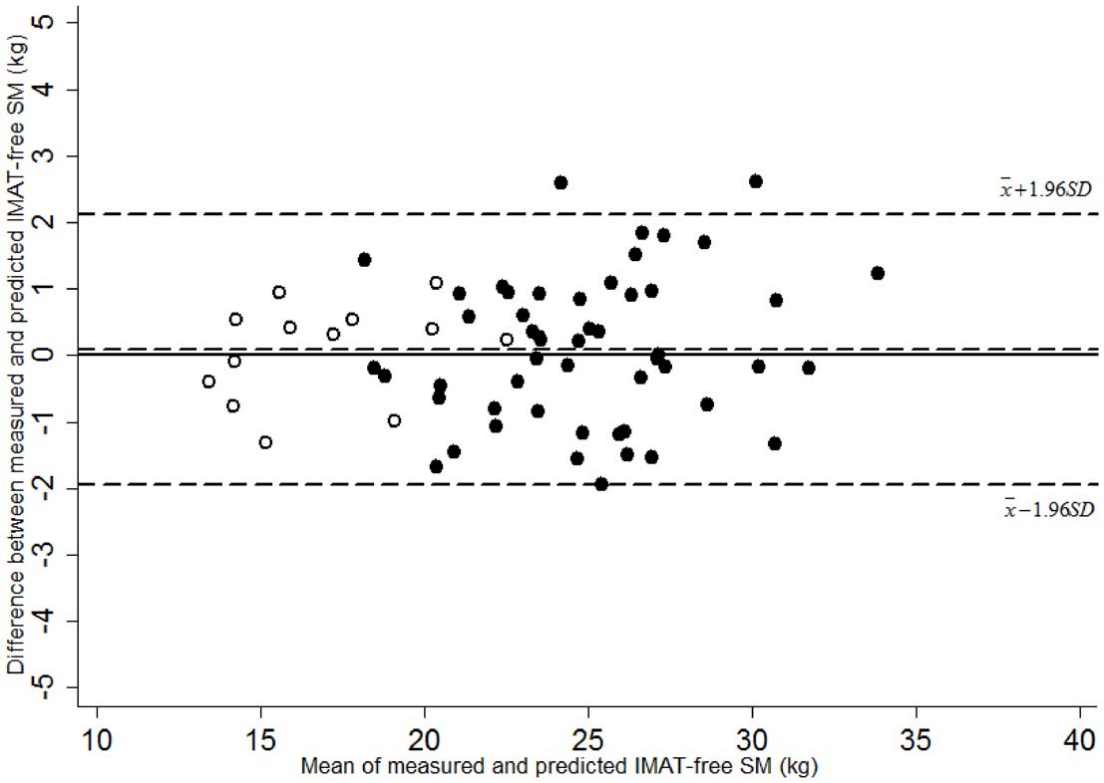
The difference between MRI-measured and DXA- predicted IMAT-free skeletal muscle (IMAT-free SM) on the ordinate versus the average of MRI-measured and DXA-predicted IMAT-free SM on the abscissa in men (•, n = 52) and women (○,n = 14) by Bland-Altman plot. Mean of measured-predicted IMAT-free SM: 0.09 kg, 95% CI -1.94 - +2.13 kg. Difference of measured–predicted IMAT-free SM = 0.03×Mean of measured and predicted IMAT-free SM –0.84, *r* = 0.10, *P* = 0.42.

IMAT-free SM predicted by both two models (model 1∶23.3±4.5 kg, model 2∶23.3±4.5 kg) were not significantly different from IMAT-free SM measured by MRI (23.4±4.7 kg) (both *P*>0.05) IMAT-free SM predicted from two models were highly correlated with IMAT-free SM measured by MRI (both *r*>0.97, *P*<0.001). The inclusion of age effect to ALST in the prediction model contributed a slight increase in the correlation between measured and predicted IMAT-free SM with *r* values from 0.972 to 0.975. There were no significant between-methods biases and the agreement of these two methods between measured and predicted IMAT-free SM by Bland-Altman analysis is presented in **Figure 6** **.**

## Discussion

The present study developed and validated DXA-SM prediction models applicable for Chinese adults. The prediction models in Chinese adults had a small but significant difference compared with prediction models for other ethnicities. The quantification of SM according to previous study underestimated SM in Chinese adults.

### Differences in IMAT, IMAT-free SM and SM between Chinese and Other Race Populations

The IMAT compartment, although comparatively small, varies in magnitude with age, sex and race [26], [27], [28], [29]. In the Chinese population, the quantities of IMAT were 0.6±0.2 kg in men and 0.5±0.1 kg in women which was comparatively smaller than the quantity of IMAT in Caucasians and African-Americans [11]. Evidence indicated that Asians have lower IMAT compared to African-Americans even after adjusting for the difference in total adiposity [26].

There was a significant difference between SM with and without IMAT in men and women (both *P*<0.001). Although there’s no obvious reduction of SEEs by removal of IMAT compared with the studies of Kim *et al.* [10], [11], when expressed relative to SM, IMAT accounted for 0.9% ∼ 5.1% of SM in the present study, and the exclusion of IMAT could eliminate the overestimation of the actual quantity of muscle. In the current study, we established models with SM and IMAT-free SM as the dependent variable with ALST and age adjusted. Kim *et al.* also extended their study by removal of IMAT in the SM compartment in diverse ethnic groups [10], [11]. Prediction models from the current study and Kim *et al.* are presented in **Table 4**. By removal of IMAT, Kim *et al.* observed lower SEEs [10], [11]. In contrast, our two models with the dependent variable as SM and IMAT-free SM have similar SEEs (1.09–1.15 kg vs. 1.05–1.11 kg), yet both have a high adjusted *R^2^* values in the range of 0.94–0.95 (**Table 3**).

**Table 4:** Prediction equations from the current study and Kim *et al.*

	Population	Gender/Age	IMAT-free SM	SM
**Present study**	**Chinese**	Age <45 years	IMAT-free SM = 1.20×ALST−0.98	SM = 1.24×ALST−1.00
		Age ≥45 years	IMAT-free SM = 1.21×ALST−0.98–0.04× (Age−45)	SM = 1.25×ALST−1.00–0.04× (Age−45)
**Kim** ***et al.*** [**10**]	**Asian**	Male	Log IMAT-free SM = 0.0189×ALST−0.0027×Age+0.0001×Age×ALST +1.1464	SM = (1.13×ALST )–(0.02×Age ) +1.58
		Female	Log IMAT-free SM = 0.0126×ALST−0.0034×Age+0.001×Age×ALST +1.1464	SM = (1.13×ALST )–(0.02×Age ) +0.97

IMAT-free SM values in Kim *et al*. were transformed using base 10 logarithms to explore the contributions of gender and race to the model.

Gender, male = 1 and female = 0.

### Why Kim et al.’s Equation Underestimates SM in Chinese Adults

We observed that, although the prediction model on SM proposed by Kim *et al.* [10] is widely accepted, there was an underestimate of SM by 2.5% in Chinese subjects when ALST was substituted into Kim’s prediction equation. The quantities of SM predicted using Kim’s equation were smaller than SM measured by MRI both in men and women (both *P*<0.05). This phenomenon even persisted in the group of subjects <45 and ≥45 years, respectively (**Table 2** **,** **Figure 3**). The observation above impelled us to explore a possible explanation. Eveleth and Tanner reported that there were considerable differences in leg lengths via the sitting height ratio among ethnic groups, with blacks having longer legs than Caucasians and Asians having shorter legs than Caucasian [30], [31]. Recent data from the NHANHS III survey confirmed that compared to Caucasians, blacks have longer legs [32] and this is also the case for a national sample of black and white youths aged 12 to 17 years old [33]. In addition, Deurenberg *et al*. reported that Asians (especially Chinese) had relatively shorter legs when compared to Caucasians [34]. Since ASM accounts for 75% of the total-body SM in the body, subjects with longer legs are expected to have more SM with the height into consideration. Previous studies have already elucidated that prepubertal Asians had less ASM than their African-American and Caucasian counterparts [35]. Although data is limited on the difference of ASM in adults among different ethnicities, Chinese adults would have less ASM compared with African-Americans and Caucasians with height taken into consideration. As a measure of ASM, ALST is a strong predictor in estimating SM by DXA. Thus comparatively, the distribution of SM in Chinese would turn out to be smaller in appendicular tissues than in Caucasians and blacks, because Chinese have the shortest legs, on average. This hypothesis might be one plausible explanation for the underestimation of Kim’s equation in Chinese adults.

It should be noted that although the general notion indicated that Asians have the shortest legs when compared to blacks and Caucasians, there were observed differences of leg length among Chinese and people from other Asian countries. Lin *et al.* reported that mainland Chinese had moderate limbs while Taiwanese had longer legs, Japanese had shorter limbs and Korean had longer upper limbs [36]. Given the existence of the discrepancy of leg length in different ethnic groups and in people from different Asian countries, the development of a specific SM-prediction model for Chinese in the present study is of vital significance and accuracy.

There was a high correlation between SM and ALST, which is consistent with previous studies [10]. In the current study, ALST alone explained 94% of the observed between-individual variation in IMAT-free SM with a low SEE (i.e. 1.11 kg), indicating high estimation accuracy in the prediction model.

### Effect of Age on SM

Our results did find that there was a curvilinear relationship between age and IMAT-free SM mass, with a change in the slope of the regression line occurring at 45 years old. The age of 45 years was thus identified as the cut-off point, after which an obvious negative association between age and SM was observed. This finding is consistent with the study of Janssen *et al.,* who reported that there were accelerating rates of SM loss in both genders in adults of multiethnic groups (67% Caucasians, 17% African-Americans, 8% Asians, and 7% Hispanics) aged 18–88 years, averaging at 45 years of age [3]. Evidence also indicated that the isometric [37], [38], [39] and isokinetic [37], [38], [39], [40] strength did not change substantially until approximately 45 years of age. Thus the age of 45 years is a critical cut-off point of total body skeletal muscle mass across lifespan.

Silva *et al.* had inconsistent findings, reporting a cut-off point of SM decline of 27 years of age, after which the SM begins to decline in a large and diverse sample [41]. Gallagher *et al*. also confirmed the decline of total body potassium, which predominantly accumulates in SM, starting from the age of 31 in men and 30 years of age in women, both African-American and Caucasian [42]. One explanation for the discrepancy of the age cut-off point when SM begins to decline between our study and the other studies by Silva *et al.* and Gallagher *et al.* is that there might be a minor decrease of SM in the third decade of adults in the present study, yet the rate of SM loss may be so slight that the decline in the quantity of SM is not noticeable. However, previous studies reported that the muscle fiber cross sectional area (i.e., contractile muscle) [43] and body cell mass [44], [45] did not change substantially until approximately at the age of 45 years. The discrepancy of observed age at which SM begins to decline may be due to ethnic differences in body composition.

### Effect of Gender on SM

The observation that men had greater SM than women agrees with the finding of previous studies [3], [10]. Because no gender difference in the relationship between IMAT-free SM and ALST was detected, we combined men and women together in the prediction model, although gender didn’t contribute to the final developed model. One possible explanation for the insignificant effect of gender in predicting SM may be attributed to the relative small sample size of female subjects, which might offset the additional prediction accuracy by this model. However, even in a previous study with moderate sample size (145 men and 176 women), gender was found to be a borderline contributor (*P* = 0.052) in the SM prediction model by Kim *et al*. [10]. On the other hand, the insignificance of gender in the prediction model might be just due to the ethnic difference *per se*. Nevertheless, the protocol to develop the prediction model may be useful when to develop similar total-body SM prediction model in a large scale of Chinese adults.

### SM/ALST Ratio

The values of IMAT-free SM/ALST in our study, 1.15 in men and 1.13 in women, were consistent with the ratios of the previous study [10]. In addition, we observed that IMAT-free/ALST decreased significantly with age in men, which is consistent with the finding of Kim *et al.* [10]. However, we failed to observe the association between IMAT-free SM/ALST with age in women.

The discrepancy of the association between IMAT-free SM and age in men and women might be due to the small sample size of women. Large scale studies are needed to explore and confirm whether IMAT-free SM/ALST decreased with age in both genders. On the other hand, because ALST is composed of muscle, skin, connective tissue, and the lean portion of adipose tissue, muscle decreases but other components such as connective tissue and the lean portion of adipose tissue increase during the aging process [10].

### Study Implications

The major contribution of this study was to establish DXA-SM prediction equations applicable for healthy Chinese adults. The DXA-SM prediction equations were developed by using MRI as the reference standard and this observation suggests that DXA can provide reliable and accurate way to estimate SM in large scale studies in Chinese adults.

In view of the strong influence of SM on bone mineral density, the observation that SM progressively decreased with age after the age of 45 years, highlights the importance of strength training, especially high-intensity strength training that can prevent osteoporosis by simultaneously promoting muscle mass, muscle strength and bone mineral density.

Sarcopenia, defined as a decrease in SM in aging men and women is of great interest. The quantification of SM is help to establish the diagnostic index of sarcopenia in the Chinese population and further explore the prevalence of sarcopenia and its health consequences.

### Study Limitations

A limitation of the present study is the relatively small size of female subjects, which may introduce a gender bias in predicting the IMAT-free SM by ALST, and it may also offset the accuracy of the prediction based on this model. Larger scale studies should be conducted in the future to explore whether there are any effects due to gender in predicting IMAT-free SM by ALST in the Chinese population.

The prediction equations developed in this study may not be appropriate for athletes and subjects <18 years. There is also a need to develop a corresponding prediction model for children and adolescents. In addition, we cannot rule out the possibility that the accuracy of this model will be compromised in subjects with disease states and conditions associated with extreme muscle atrophy, such as spinal cord injury.

### Conclusions

In conclusion, SM predicted according to a previous model developed in multi-ethnic groups was underestimated by 2.3% and 3.4% for Chinese men and women, respectively. A new prediction equation by DXA has been established to estimate IMAT-free SM in Chinese adults. This observation suggests that DXA is a reliable and accurate approach to study the quantity of total body SM and its association with age and gender on a large scale in Chinese populations.
